# “Telling” and assent: Parents’ attitudes towards children's participation in a birth cohort study

**DOI:** 10.1111/hex.12630

**Published:** 2017-09-22

**Authors:** Izen Ri, Eiko Suda, Zentaro Yamagata, Hiroshi Nitta, Kaori Muto

**Affiliations:** ^1^ Graduate School of Interdisciplinary Information Studies The University of Tokyo Tokyo Japan; ^2^ National Institute for Environmental Studies Tsukuba Japan; ^3^ Department of Health Sciences Interdisciplinary Graduate School of Medicine and Engineering University of Yamanashi Chuo Japan; ^4^ Department of Public Policy The Institute of Medical Science The University of Tokyo Tokyo Japan

**Keywords:** birth cohort study, children, informed assent, parents, qualitative research, telling

## Abstract

**Introduction:**

One of the ethical issues surrounding birth cohort studies is how to obtain informed assent from children as they grow up. What and how parents tell their children affects children's future choices about the study, yet few studies have focused on parents’ influence on children.

**Objective:**

This study examines parents’ attitudes towards telling their children about their participation in a specific birth cohort study.

**Methods:**

We conducted surveys and in‐depth interviews with the parents of children who participated in the “Japan Environment and Children's Study” (JECS), which follows children from the foetal stage to age 13.

**Results:**

Forty‐four mothers and 23 fathers answered the survey, and 11 mothers and 3 fathers participated in in‐depth interviews. Parents’ attitudes towards “telling” were categorized into 3 communication styles depending on their perception of the risk/benefits for their children. Most parents predicted that the study would benefit their children and preferred “directive telling,” which we divided into “empowered telling” (provides children with a positive identity as participants) and “persuasive telling” (attempts to persuade children even if they express reluctance as they grow). A few parents, weighing the study's potential risk, preferred “non‐directive telling,” which respects children's choices even if that means withdrawing from the study.

**Discussion:**

While “directive telling” may lead children to have positive associations with the study, children should also be told about the risks. Investigators can provide materials that support parents and give children age‐appropriate information about their participation, as well as ensure opportunities for children to express their feelings.

## 1 INTRODUCTION

### 1.1 Parent's proxy consent and children's informed assent in birth cohort studies

Several countries have implemented longitudinal birth cohort studies; national projects in UK have been going on since the 1940s (ie, the 1946, 1958 and 1970 Birth Cohort, and 2000 Millennium Cohort Study), and recently, Denmark, Norway and Japan have implemented their own large‐scale studies, each with around 100 000 participants. These studies usually follow children from the foetal or neonatal stage until they are teenagers or adults and study the possible long‐term health consequences of maternal lifestyle, genetic factors and early exposure to environmental contaminants and other substances.^1^, ^2^

Key ethical issues in birth cohort studies involve the following: (i) parents’ initial proxy consent, which is related to recruitment and enrolment, and (ii) investigators’ subsequent obtaining of informed assent—defined as “affirmative agreement to participate in research”—from those children capable of providing it.^3^ While pregnant mothers may give consent by proxy, once children become older it is ethically important to provide them with the opportunity to express their own opinions and involve them in the decision‐making process, depending on their level of comprehension.^4^, ^5^, ^6^ Moreover, after children mature, investigators must again obtain informed consent from adolescents.

### 1.2 Parents’ influence on children's decision making in birth cohort studies

There have been studies regarding parent's proxy consent, most of which focus on pregnant women's incentives/disincentives to give consent, and attitudes towards children's future participation in birth cohort studies.^7^, ^8^, ^9^, ^10^ Garg et al^10^ show that some mothers demonstrate discomfort when making proxy consent on behalf of their children and voice concern over possible psychological harm, anticipating their children's feelings as they mature. Regarding children's assent, Goodenough et al^11^ interview children about their perceptions of participating in a specific birth cohort study (the Avon Longitudinal Study of Parents and Children: ALSPAC, UK) and find that some children as well as parents misinterpret their involvement or role in the study.

Although these studies clarified pregnant mothers’ attitudes and children's perceptions towards the studies, fewer studies focus on “informed” stages of assent, or the process by which children are given information about the study in which they participate. Helgesson^12^ raises a question of “how to inform children” and suggests that information should be shared in advance of their capacity for full decision making in ways that extend beyond verbal descriptions: drawings, photographs or video recordings.

Besides media or communication tools, *who* shares information with children is also important. When the participants are adults, informed consent must be sought by an individual who has no prior relationship with the study participants;^13^ such roles are usually expected to be performed by clinical research coordinators, research nurses, counsellors or other health‐care professionals.^14^, ^15^, ^16^

When the participants are children, as in birth cohort studies, parents—who often gave consent by proxy before or shortly after the child is born—may be involved in the explanation process before investigators or health‐care professionals seek informed assent. Since children are especially susceptible to parents’ influence, children's decisions in this case cannot be understood as autonomous, but rather as a transformation of the parent‐child interaction.^17^

We can assume that at some point, children will be told that they were recruited into a longitudinal cohort study. How children learn this information may influence their attitudes towards the study and their future decisions concerning whether to continue cooperating. However, despite the importance of how children are informed, little work has been done on parents’ role and influence on their children's decision making.

### 1.3 “Telling” as the premise for informed assent

Based on the above information, we propose a framework for parents to convey information to their children called “telling,” which was originally used for adopted and donor‐conceived children. These children have the “right to know” their origin, and parents’ ability to “tell” their family story is a way for children to learn the information in an age‐appropriate manner. Parents are increasingly recommended to openly talk with their children,^18^, ^19^, ^20^ and the process of “telling the story” about a child's birth is an ongoing one that starts when children are very young and is repeated daily as they grow older.^21^

The same method of “telling” can be applied to birth cohort studies: this is another situation in which children have to be told something that was already determined before their birth. As in the case of adoption, parents should be the initial storyteller, prior to interactions or conversations with physicians/investigators.

Thus, the purpose of this study was to confirm parents’ attitudes towards “telling” their children about study participation in a birth cohort study. This article presents our qualitative analysis of parents whose children participated in the “Japan Environment and Children's Study” (JECS), a large‐scale, nationwide birth cohort study which we will later describe in detail.

## 2 METHODS

### 2.1 Case study

The Japanese Ministry of the Environment funded a large‐scale, nationwide birth cohort study in 2011. Called the “Japan Environment and Children's Study” (JECS), it follows 100 000 children from pre‐birth to age 13.^22^ Fifteen Regional Centers across Japan were responsible for recruitment and participant follow‐up, and from January 2011 to March 2014, they recruited women in the stages of early pregnancy at obstetric facilities and/or local government offices that issued mother‐child health handbooks.^23^

The 2015 “Ethical Guidelines for Medical and Health Research Involving Human Subjects” enforced by the Japanese government requires investigators to endeavour to obtain informed assent from minors under 16 years old who are considered capable of expressing their intentions.^24^ The JECS is the only nationwide birth cohort study conducted since the rule were put in place. Parents were informed when they gave initial consent that, at some point in the research process, children would be asked to confirm their intentions to continue as research participants.

### 2.2 Study area and design

We conducted our research with “JECS Yamanashi,” located at the Koshin‐Yamanashi Regional Center (University of Yamanashi) in the Yamanashi prefecture, which itself is located near the centre of Honshu (the main island of Japan), 2 hours’ drive from Tokyo. The participants of JECS Yamanashi were recruited from 5 cities: Kofu, Chuo, Koshu, Yamanashi and Fujiyoshida. Study participants included 4474 children, 4630 mothers and 3040 fathers.^25^ At the time, the entire population of the Yamanashi prefecture was 857 690, with a 2011 total number of live births of 6412;^26^ participants thus included over 50% of newborns in the 5 cities during the 3‐year recruitment period. Besides the investigators, there were 7 research coordinators involved in the study who were in charge of recruitment, collecting questionnaires, conducting health check‐ups, monitoring air quality and managing participant events.

We adopted a mix of quantitative and qualitative methods in this study; we conducted a small, face‐to‐face survey and followed this with in‐depth interviews. The survey mainly consisted of closed‐ended questions, which allowed us to comprehensively collect answers. Using the data collected from our survey, we next conducted in‐depth interviews, which consisted of open‐ended questions, in order to better understand the reasons for respondents’ answers. To find participants for the interviews, we handed invitation letters to respondents and later followed up with both them and some of their partners. We adopted a “maximum variation sampling” strategy^27^, ^28^ in order to include a wide range of extremes in demographics (including occupation, number of children, children's ages and children's genders), motivations for participating and degree of interest in the JECS.

Our research proposal was approved in advance by the research ethics committees of the University of Tokyo, the University of Yamanashi and the National Institute for Environmental Studies (NIES).

### 2.3 Data collection

Three interviewers (IR, ES and KM) conducted the face‐to‐face survey at the event booth for JECS Yamanashi participants during community events on 22 and 23 August 2015. Each survey took about 10 minutes, and we asked 17 closed‐ended questions and 2 open‐ended questions on topics such as parents’ motivations for participating in the JECS and their attitudes towards telling their children. We then recorded the answers on questionnaire forms. We surveyed mothers and fathers separately, although we numbered the questionnaires to reciprocally compare couples’ answers in our analysis.

One of 2 interviewers (IR and/or ES) carried out each in‐depth interview between October and November 2015. Interviews took place either in a University of Yamanashi meeting room or in participants’ homes. When both parents of a child were interviewed, the mother and father were interviewed individually. We obtained written informed consent from each interviewee. Each interview took about 1.5 hours, and they were all recorded. We asked 20 open‐ended questions on a variety of topics, including parents’ motivations for participating in the JECS; who held the major responsibility for childcare; whether they had talked about the JECS with their partner, family or friends; and their attitude towards providing personal information. We also provided information about the JECS study schedules for the next 13 years and about the new informed assent rule, which enabled parents to imagine what and how they would tell their child about their study participation at each development stage, as well as how their child would react.

### 2.4 Data analysis

Survey data were simply aggregated, and transcripts of the in‐depth interviews were coded using Qualitative Data Analysis Software MAXQDA10 (VERBI GmbH). IR generated most of the codes and categories and held several meetings with ES and KM to verify the coding. The final data analysis examined the following categories: parents’ attitudes towards the necessity of disclosing study participation to their child, parents’ willingness to disclose, who and with whom parents planned to tell, what and how to tell their children and various background information, including why parents participated in the research and what kind of benefits they thought this would give them and their children. Parents’ communication style emerged though the analysis, and this article categorizes these styles into “telling” categories and elaborates on each.

## 3 RESULTS

### 3.1 Sample characteristics

In total, 44 mothers and 23 fathers (including 22 couples), whose children were aged approximately 9 months to 4 years, participated in our survey (Table 1). About half of the fathers were also JECS participants. Eleven mothers and 3 fathers participated in our in‐depth interviews (Table 2). All 3 fathers we interviewed were also JECS participants and the partners of women who were also interviewed. Their children were aged from 10 months to 3 years, 3 months.

**Table 1:** Sample characteristics of survey

Mother (N = 44)^a^	n (%)	Father (N = 23)^a^	n (%)
Maternal age		Parental age	
<25	0 (0.0)	<25	1 (4.3)
25‐29	8 (18.2)	25‐29	4 (17.4)
30‐34	14 (31.8)	30‐34	7 (30.4)
35‐39	14 (31.8)	35‐39	5 (21.7)
40‐44	7 (15.9)	40‐44	4 (17.4)
≥45	1 (2.3)	≥45	2 (8.7)
Partner's study participation		Study participation	
Yes	29 (65.9)	Yes	15 (65.2)
No	14 (31.8)	No	8 (34.8)
Don't Know	1 (2.3)	Don't Know	0 (0.0)
Child's age^b^		Child's age^b^	
<1	5 (11.3)	<1	2 (8.7)
1‐2	24 (54.5)	1‐2	14 (60.9)
2‐3	8 (18.2)	2‐3	4 (17.4)
3‐4	6 (13.6)	3‐4	3 (13.0)
≥4	1 (2.3)	≥4	0 (0.0)
Child's gender^b^		Child's gender^b^	
Male	20 (45.5)	Male	12 (52.2)
Female	24 (43.6)	Female	11 (47.8)
	44 (100)		23 (100)

a There were 22 couples.

b The population includes parents who have twins.

**Table 2:** Sample characteristics of in‐depth interviews

ID	Age	Child's age	Child's gender	Partner's study participation	Occupation
Mother 1	35	0 y 10 mo	Male	Yes	Housewife
Mother 2	33	1 y 6 mo	Female	Yes	Housewife
Mother 3	33	3 y 3 mo	Female	No	Housewife
Mother 4	26	1 y 10 mo	Male	Yes	Childcare leave
Mother 5	31	1 y 7 mo	Male (twins)	No	Housewife
Mother 6	37	2 y 0 mo	Female	Yes	Temporary worker
Mother 7^a^	39	2 y 3 mo	Female	Yes	Housewife
Father 7^a^	39			Yes	Full‐time worker
Mother 8^a^	36	3 y 1 mo	Female (twins)	Yes	Full‐time worker
Father 8^a^	38			Yes	Full‐time worker
Mother 9^a^	39	2 y 3 mo	Female	Yes	Housewife
Father 9^a^	32			Yes	Self‐employed worker
Mother 10	25	1 y 6 mo	Female	Yes	Housewife
Mother 11	40	1 y 3 mo	Male	Yes	Part‐time worker

a Mother and Father 7, 8, 9 are couples, and they were all interviewed separately.

### 3.2 Reasons for participating in the study

Parents described various motivations for participating in the study. Common reasons included free health check‐ups, an interest in the JECS study, a desire to do something good for their child and hope that the study would solve an environmental or health problem. It is noteworthy that several informants mentioned that they participated in the JECS because “I just happened to be recruited in the hospital” or “I was talked into it by a midwife.”

Since recruitment and consent occurred during pregnancy, interaction with the research coordinators, some of whom were also midwives or public health nurses, was perceived as helpful for the pregnant mothers. Some mothers expressed their gratitude for the support they received at the hospital:

It was important for me to be told ‘You'll be all right’ by the research coordinator during the time (pregnancy)… I was relieved to hear that before giving birth, and my baby was born safely, so I decided to participate in the study. [Mother 8, 36, female (twins), 3 years 1 months]

Face‐to‐face communication before a child's birth seemed to build friendly relations between mothers and research coordinators, and such interaction sometimes continued even after the child was born. Parents were pleased that the research coordinators still remembered them when they met again at events, which eventually led investigators and the JECS study to gain more credibility in parents’ eyes.

### 3.3 Personal benefits of participation for parents and children

Our informants discovered that there were various advantages to participating in the study, both for themselves and for their children: parents received blood tests and could access some of their results (ie, allergy testing), they considered their children's growth and reflected on everyday habits through answering questionnaires, families were sent newsletters with interesting statistical data or useful information about health and the environment, and there was some monetary compensation or small gifts for their participation. Many family members, especially mothers, had many favourable comments regarding the events held by JECS Yamanashi. For mothers, these events were not only fun, but also a way to build a community among the “JECS moms.” Some informants even said they were reluctant to talk about the JECS with mothers who did not participate, saying that “those who did not have rights to participate” would “envy me,” since enrolment was limited to a certain time period and geographical area.

In general, a longitudinal observational study such as the JECS has no direct benefit to the children participating. Compare this with a clinical study, which, in some cases, will benefit the patient. However, our informants perceived that the JECS study would have benefits not only for “future children,” but also their own:

I hope that the JECS study will be useful for future children. She is my first child, so I wanted to do something for her. I hadn't done such a thing before, and I thought that it was a very good idea. [Mother 3, 33, female, 1 year 6 months]

The most important reason I contributed is social contribution. It may be difficult for adults to take a first step to do something, so it is better to get a start now when my kid is young. [Father 9, 39, female, 2 years 3 months]

Some parents hoped that becoming a member of a national study would be a good opportunity for their children to foster altruism. They seemed to find significance in the educational value of study participation.

### 3.4 Parents’ attitudes towards “telling”

In our survey (N = 44 for mothers, N = 23 for fathers, including 22 couples), 72.7% of mothers and 60.8% of fathers believed it was necessary to tell children of their participation in the research study. Although no parents had told their children at the time of the interviews, 81.8% of mothers and 43.4% of fathers said they would tell their children at some point in the future. 38.6% of mothers and 43.5% of fathers preferred to tell the children with their partners or family, and 25.0% of mothers and 8.7% of fathers preferred to tell them on their own. In contrast, only 15.9% of mothers and 13.0% of fathers preferred to have professionals get involved in the early stages of telling. Therefore, it seems that they valued communication within the family. In general, mothers had positive and active attitudes towards telling their children, while fathers showed more unmotivated attitudes or expected their partner (both as children's mothers and as the JECS participants) to play major roles in telling. When comparing the answers of the 22 couples, 8 couples stated a different preference for whether to tell their children and who would be responsible for doing so.

### 3.5 What and how to tell? Three communication styles

One of the major questions our study tried to answer is how parents planned on telling their children about participation in the study. Although none of the parents had told children yet, since they were too young at the time, the interview process led them to imagine their grown‐up children and anticipate the long‐term future. By analysing the recorded narratives, we found that communication styles seemed to vary depending on parents’ perception of the JECS study's risks and benefits. We categorized their answers into 3 communication styles. Twelve parents, who predicted that the study would benefit their children, preferred “directive telling,” which we further divided into (i) “empowered telling”, and (ii) “persuasive telling.” Only 2 parents, who weighed the possible benefits with the potential risk, preferred (iii) “non‐directive telling.”

#### 3.5.1 Empowered telling

“Empowered telling” emphasizes the idea that the study will have benefits and encourages children to continue in the study, providing them with a positive identity as participants.

I want to tell my children, “It will be helpful not only for others, but also for you.” If my kids take care of their children, or have jobs or academic study related to children, participating in the JECS may give them an advantage. [Mother 8, 36, female (twins), 3 years 1 months]

Mother 8 expected that her twin daughters might become mothers themselves someday, and she intended to emphasize the fact that the study would help them in the future so her children would accept it positively. As many parents perceived that it was difficult for children to understand altruism, they planned on telling them not only “you are helping others,” but also “this study is helping you.”

As the next quotation by Mother 3 shows, other narratives based the benefits around having some kind of “special privilege.”

I may say to my daughter, “Only people who lived in a certain region could participate in this study, and this city was chosen by chance, so you are lucky to join it”…

Or I can say to her, “You are the chosen one,” and she will be proud. [Mother 3, 33, female, 3 years 3 months]

The enrolment period for the JECS study lasted from January 2011 to March 2014, and recruitment was limited to certain cities. Although expectant mothers in the area were exhaustively recruited, Mother 3 felt that her daughter was “lucky” to participate, and hoped she would be proud to be a member of this national project.

Parents often preferred to use such empowered telling techniques in order to build a positive identity around being a participant so that children would happily agree to continue participating. Rather than being a one‐shot discussion, these parents supported telling children about the study early and often, according to their age and level of understanding.

If he understands it's valuable to take part in the study he's been involved in since childhood, he may not quit. I plan to take a step and tell him the difficult things gradually: dividing the conversation into many when he is 2 years old, 3 years old, in kindergarten, and elementary school‐aged. Only once or twice may be insufficient. [Mother 1, 35, male, 0 year 10 months]

#### 3.5.2 Persuasive telling

Parents who preferred a “persuasive telling” strategy avoided sharing negative information that might lead to their child's refusal. Their goal was not only to empower their child, but also to persuade them to continue participating, even if they were unwilling.

I want to tell them why and how they participated in the JECS when they can understand, and encourage them by saying something like, “Why don't you continue, anyway?” If something happens, they will quit the study. So, I'll explain so they don't quit. [Mother 8, 36, female (twins), 3 year 1 months]

Mother 8 strongly hoped her children would continue participating in the JECS study. She anticipated that if her children were suddenly told about the study when they got older, they would be confused and become reluctant, so she planned on providing them with visual materials—such as graphs or pictures that show the study data—and newsletters. This way she could present their contribution in a way that could be easily understood by children. She articulated that she would make plans in advance so as “not to be refused by my kids.”

All these parents agreed that they would first ask their children why they were reluctant, then try to lighten the burden and let them take a break from the study for a while and finally “respect the child's intention” if they had convincing reasons to withdraw without compelling them to continue. Despite respecting children's autonomy, no parent thought that persuading them would be impossible.

For example, I'll say, “You've done it since you were a child, and it will be completed in just a bit longer, so let's keep at it.” [Mother 4, 26, male, 1 year 10 months]

I think she should think twice. It will be a waste to quit when you already have participated for so long. [Mother 6, 37, female, 2 years 0 month]

If she wants to withdraw because she just wants to play, I'll explain properly and tell her she is being helpful by responding the study questionnaires. [Mother 9, 39, female, 2 years 3 months]

These parents repeatedly emphasized that their children were being “helpful” for the JECS study which would be also “helpful” for their children; they planned to use dialogue to try to persuade their children to continue. Interestingly, as the quote by Mother 4 shows, parents plan to continue to talk to and persuade their children as they get older; the study is scheduled to end when children reach age 13. It seems that some parents want their children to participate regardless of their level of understanding and expect them to finish the study even if they express refusal, especially if they have already participated in the study for a long period. These continuation‐oriented parents seemed to hope to teach their children values like “diligence” or “altruism” through study participation.

#### 3.5.3 Non‐directive telling

In contrast to “directive telling” methods like “empowered telling” and “persuasive telling,” parents who preferred “non‐directive telling” considered the possibility their child would, at some point, refuse to contribute to the study. They tried to respect their children's intentions and choices, even if this meant withdrawing.

If the results of the JECS study were already utilized now, it would be easy to tell my child, but it is not yet, is it? How can I explain things that are still being worked on? I think that it is difficult to state the goal when the goal is yet in the future. [Mother 11, 40, male, 1 year 3 months]

Mother 11 was sceptical about the benefit of the study for her child and perceived a risk in providing her child's personal information. She thought the only way to persuade her son would be to show him concrete results and that children would not be convinced if the study turned out to be useless. She did not believe that children under 13 had the ability to comprehend the objectives or details of the study. Nevertheless, if privacy breach from the JECS should be revealed, she would permit her young son to withdraw from the study because “It’s [my] children’s information.” She said that she felt responsible for giving proxy consent and providing her son’s personal information.

Similarly, despite expressing doubt about the children's ability to comprehend when they are under 13 years old, Father 7 intended to tell his daughter about why he chose to participate in the study, the study's objectives, and how her information was used.

The big issue is whether to disclose the data to the children. For example, the study ends when they are 13 years old, and then they are told about it. They may just say, “Hmm, that's OK,” but they also may say, “So, how is the data going?” I really can't say. [Father 7, 39, female, 2 years 3 months]

Father 7 seemed to value accountability to children and anticipated that his younger daughter would wonder, “Why do I have to do things that my brother doesn't?” He considered the possibility of sharing data with his child and wanted to respect his daughter's choices. Unlike Mother 11, he perceived the benefits his family would receive and hoped his daughter would understand the value of the study. However, he did have concerns about giving one's whole life log to the JECS.

## 4 DISCUSSION

In this study, we clarified parents’ attitudes towards telling their children about participating in the JECS study. Previous studies paid little attention to the role of parents in providing information or the impact on children. Our results suggest that parents are willing to provide their children with positive information about the main aspects of the study and to empower or sometimes persuade them to continue, even though they are under no obligation to do so. Although parents’ narratives in this study were based on imaginary dialogue with their future children, we suspect that the reason parents prefer “directive telling” to “non‐directive telling” is related to their own feelings towards the JECS study. Previous studies show that participants’ primary motivation for taking part in clinical trials is their own personal benefit, which is not directly related to health outcomes.^29^ Results indicate the same is true for observational studies. Most parents perceive the JECS will not only benefit “future children's health,” but also their children's. Our results in this survey also generally support a previous questionnaire survey, which asked participants in another Regional Center why they chose to participate in the JECS.^30^

On the other hand, a few parents believed the possible risks or burdens outweighed the benefits. They had concerns about the leak of personal information and giving one's whole life log to the JECS. It is important to note that such parents believed that the information belonged to their children, not themselves. Garg et al^10^ also note that some pregnant women were concerned about the potential risks and psychological harm to children, although such views were not dominant in our participants.

Interestingly, it seemed that mothers’ and fathers’ attitudes were not necessarily consistent. For instance, while Father 7, who preferred “non‐directive” telling, was sensitive to data use and sharing and anticipated his daughter would have doubts about her study participation, his partner, Mother 7, was optimistic and perceived there to be little potential risk. Our survey showed that mothers who gave proxy consent were more actively engaged in the study and had more interaction with research coordinators. In contrast, fathers were less involved in the study or paid less attention to how to tell their children in the future. The effect of gender differences on study awareness and expectations regarding family parental roles may be another issue at hand, though it is beyond the scope of this article.

Our survey and in‐depth interviews showed that it is possible that parents did not fully understand or remember the details of giving consent and thus were not fully aware of the study schedule or what their children would experience in the study until age 13. Some of our informants (Mother 3, Mother 5 and Mother 10) had only a vague memory of what was explained in their written consent materials. As the study goes on, parents may have an increased difficulty in explaining to their children the upcoming study schedule, use of recorded data and possible risks, as the problem of participants’ limited knowledge and understanding has been pointed out in other studies.^11^, ^12^

Although we are uncertain of what parents who prefer “directive telling” will eventually say to their children in future, we might anticipate that they will convey mostly positive information about the study. Such a long communication process that continues as children develop may eventually lead these children to continue participating. For longitudinal cohort studies, it is crucial to maintain a high follow‐up rate and minimize withdrawals, so “directive telling” may be effective to accomplish the study. It has been pointed out that physicians and investigators tend to presume a child's incompetence and that what children are told often affects their ability to make a choice about participation in such a study.^31^ However, investigators also have an ethical duty to respect participants’ wishes. They should be conscious of power relationships between adults and children and be alert to the possibility of parents addressing excessively “directive” messages to their children.

Therefore, “non‐directive telling” is also an important part of protecting child participants. A feasible approach to educating children and enabling them to give assent may be to provide parents with materials like picture books that can be easily understood by children. This enables them to learn age‐appropriate information at each step. Such materials give both mothers and fathers a way to explain difficult details or descriptions and also remind them of the purpose and goals of the study.

In addition, since children's understanding and co‐operation are vital for the study to be successful, investigators should work directly with children as they get older. As children grow, the nature of the questions in the survey will change from what may be answered by parents—topics like breast‐feeding, baby food, or speech delays—to questions that must be answered by children themselves—school, bedtime or more sensitive questions like the development of secondary sexual characteristics during puberty. Since children's information or biological samples are accumulated and stored based on parental consent, some children may feel confused or uncomfortable when they are told about participation in the study. Thus, the psychological burdens on children and the burden of their decision of whether to continue or withdraw from the study should also be taken into consideration, even when parents tell them that they can withdraw from the study at any time. Investigators should make sure that children are provided with the opportunity to express their feelings or “dissent,” regardless of their ages.

### 4.1 Study limitations and future directions for research

There were some limitations in our sampling, including the method and the number of informants. Quantitative research with a large number of participants from other Regional Centers is needed to verify our pilot qualitative research; this will also allow us to consider parents’ attributes, the differences between mothers and fathers, and children's gender. We also have to consider the social and cultural context, since parent‐child relationships may be different in Japan than in other countries. Similarities and differences in parental views or education policies should be explored though an international comparative study of birth cohort studies. The next question would be whether and how investigators should try to speak with children independently from parents when the children grow up.

The most crucial point is that since the children were so young when we conducted our research, we were unable to discuss any actual “telling” experiences; future research that explores how parents actually tell their children is vital for testing our temporary hypotheses. One of the important ethical and legal considerations in the JECS protocol is its policy informed assent; the following agenda may be used to conduct additional research regarding participants’ attitudes and “telling” strategy over the long‐term. Children's attitudes must be respected and investigated;^11^, ^32^ it is important to investigate how children act on information and the changing dynamics between parents and children as they mature.

## CONFLICT OF INTEREST

The authors declare no conflict of interest.
